# DNA strand breaks induced by nuclear hijacking of neuronal NOS as an anti-cancer effect of 2-methoxyestradiol

**DOI:** 10.18632/oncotarget.3913

**Published:** 2015-05-06

**Authors:** Magdalena Gorska, Alicja Kuban-Jankowska, Michal Zmijewski, Antonella Marino Gammazza, Francesco Cappello, Maciej Wnuk, Monika Gorzynik, Iwona Rzeszutek, Agnieszka Daca, Anna Lewinska, Michal Wozniak

**Affiliations:** ^1^ Department of Medical Chemistry, Medical University of Gdansk, Gdansk, Poland; ^2^ Department of Histology, Medical University of Gdansk, Gdansk, Poland; ^3^ Department of Experimental Biomedicine and Clinical Neurosciences, Section of Human Anatomy “Emerico Luna”, University of Palermo, Palermo, Italy; ^4^ Euro-Mediterranean Institute of Science and Technology, Palermo, Italy; ^5^ Department of Genetics, University of Rzeszow, Rzeszow, Poland; ^6^ Department of Pathophysiology, Medical University of Gdansk, Gdansk, Poland; ^7^ Department of Pathology and Experimental Rheumatology, Medical University of Gdansk, Gdansk, Poland; ^8^ Department of Biochemistry and Cell Biology, University of Rzeszow, Poland

**Keywords:** 2-methoxyestradiol, neuronal nitric oxide synthase, reactive nitrogen species, nitric oxide, osteosarcoma

## Abstract

2-Methoxyestradiol (2-ME) is a physiological metabolite of 17β-estradiol. At pharmacological concentrations, 2-ME inhibits colon, breast and lung cancer in tumor models. Here we investigated the effect of physiologically relevant concentrations of 2-ME in osteosarcoma cell model. We demonstrated that 2-ME increased nuclear localization of neuronal nitric oxide synthase, resulting in nitro-oxidative DNA damage. This in turn caused cell cycle arrest and apoptosis in osteosarcoma cells. We suggest that 2-ME is a naturally occurring hormone with potential anti-cancer properties.

## INTRODUCTION

2-Methoxyestradiol (2-ME) is a physiological metabolite of 17β-estradiol (E2). It is synthesized through the hydroxylation and *O*-methylation of E2 at the 2-position [1, 2]. Serum levels of 2-ME range from 3 × 10^−11^ M in men to as much as over 3 × 10^−8^ M in pregnant women [3–6]. Pharmacological concentrations of 2-ME (10^−7^ – 10^−5^ M) inhibit cancer in various experimental models. *In vitro* and *in vivo* models revealed that 2-ME inhibited growth of colon [7], breast [8, 9], lung [10] cancer as well as endothelial cells [11, 12]. 2-ME (branded as Panzem) is currently recognized as a potent inhibitor of angiogenesis and tumor growth [7, 13–17]. Recommended oral dose of 2-ME is 1000 mg, 4 times a day [14]. Treatment with 2-ME NanoCrystal dispersion enhanced clinical benefit rate due to the improved bioavaibility of the compound and is preferably used in clinical practice [14, 15]. Steady-state C_max_ plasma concentration of 2-ME reached a pharmacological concentration of 2.17 × 10^−7^ M. The minimum estimated target concentration of 2-ME is 1.1 × 10^−8^ M, which is considered as a high physiological concentration [13, 14]. Multiple clinical trials have used 2-ME as an efficient therapeutic agent for several types of cancer [7, 13–17]. In contrast, there are only a few studies concerning the physiological activity of 2-ME [5, 6, 53]. In spite of its proven anticancer activity, the molecular mechanisms of 2-ME remain unclear. Preclinical studies suggest that 2-ME directly inhibits angiogenesis and induces apoptosis in tumorous and rapidly proliferating cells. 2-ME induces both extrinsic and intrinsic apoptotic pathways associated with the overexpression of p53 [18, 19, 20]. Additionally, it takes part in stress-induced apoptosis due to the generation of reactive oxygen (ROS) and nitrogen (RNS) species [21–23]. Our previous study demonstrated that the anticancer effects of 2-ME are associated with the selective increase in neuronal nitric oxide synthase (nNOS) within highly metastatic osteosarcoma (OS) 143B cells [21]. In 2002, Su and co-workers reported that microtubule-disturbing agents, including 2-ME, modify NO generation [24]. Nitric oxide synthases (NOSs) are a group of hemoproteins that catalyze the oxidation of L-arginine to citrulline, releasing a molecule of nitric oxide NO (II) [25]. At least 3 isoforms of NOS have been distinguished: neuronal nitric oxide synthase (nNOS, NOS 1, NOS I), found mainly in neurons; inducible nitric oxide synthase (iNOS, NOS 2, NOS II), induced by factors such as stress or inflammation; and endothelial nitric oxide synthase (eNOS, NOS 3, NOS III), expressed mainly in endothelial cells [25]. The regulatory mechanisms controlling the expression and localization of nNOS are very complex. Though nNOS is usually found within the cytosol, it may be also recruited to the nucleus [26, 27, 28]. The reasons for the nuclear recruitment of nNOS remain unclear.

In our study, we investigated the anticancer effects of 2-ME at physiologically and pharmacologically relevant concentrations in osteosarcoma (OS) cell models. OS is one of the most common bone cancers of childhood and adolescence. It is characterized by the formation of immature bone structures or osteoid tissue by cancerous cells [29, 30, 31]. In the light of many studies, 2-ME can become a potent and relatively safe treatment for OS patients [19, 32, 33, 34, 35]. Here, we showed that the anticancer properties of 2-ME may be explained by DNA damage caused by generation of nitric oxide (NO). 2-ME increased nuclear localization of nNOS in OS cells, possibly causing nuclear NO production. Thus, 2-ME could be considered as a naturally occurring hormone of potential oncostatic properties.

## RESULTS

### Effect of physiological and pharmacological relevant concentrations of 2-ME on OS 143B cell death

**Figure 1:**
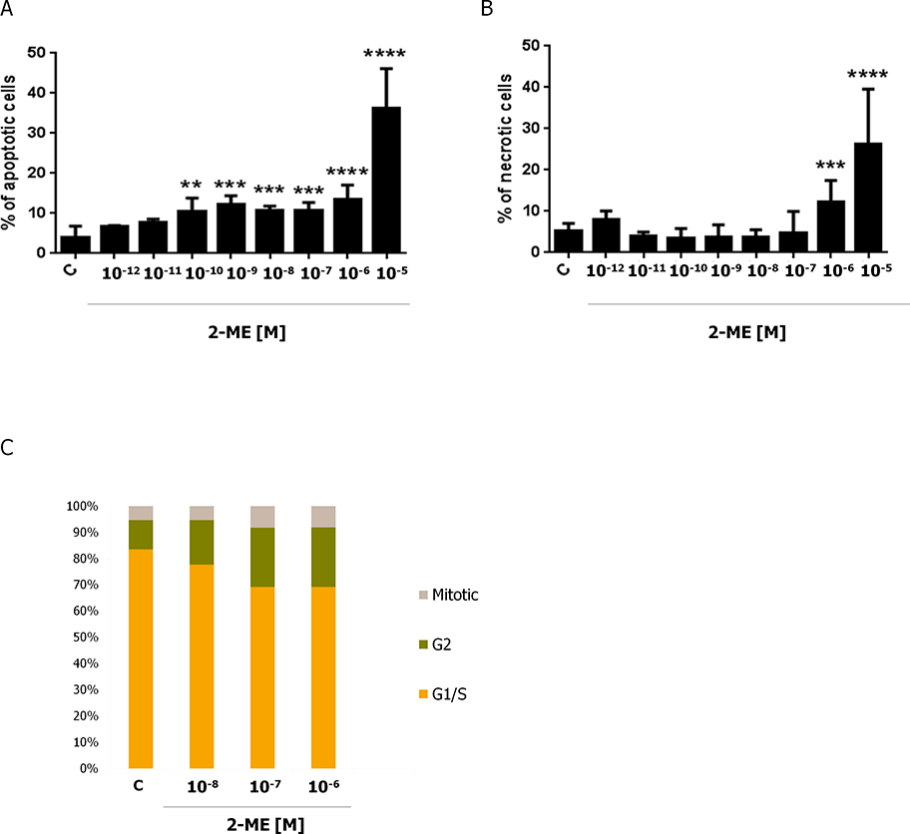
Induction of cell death and inhibition of cell cycle of 143B cells by 2-ME Induction of apoptosis **A.** and necrosis **B.** by 2-ME. 143B OS cells were treated with different concentrations of 2-ME (10^−12^ M – 10^−5^ M) for 24 h. The cells were then harvested and the percentage of apoptotic and necrotic cells was determined by Annexin V-PI staining. Values are the mean ± SE of three independent experiments (*N* = 6 replicate cultures). The absence of an error bar denotes a line thickness greater than the error. *P < 0.01, **P < 0.001, ***P < 0.0001, ****P < 0.00001 versus control cells (C). **C.** Cell cycle analysis in 143B OS cells treated with 2-ME. After a 24 h treatment with 2-ME (10^−8^ M – 10^−6^ M), the cell cycle arrest was determined using the In Cell Analyzer 2000. The cells were stained with a mixture of Hoechst 33342 and Cell Trace™ Calcein Red-Orange. Each experiment was performed at least three times.

Our first goal was to determine the influence of physiological (10^−12^ M – 10^−8^ M) and pharmacological (10^−7^ M – 10^−5^ M) relevant concentrations of 2-ME on induction of cell death within 143B OS cells. These concentrations were determined from the available literature data [3–6, 19, 21, 33, 42–47]. Previously, we demonstrated that 2-ME inhibited cell growth and induced cell death in hippocampal (HT22) and OS (143B) cell lines at high pharmacological concentrations [21]. Herein, the cells were treated with different concentrations (10^−12^ M – 10^−5^ M) of 2-ME for 24 h. Induction of apoptosis and necrosis was determined by flow cytometry. 2-ME induced apoptosis in 143B OS cells not only at tested pharmacological relevant concentrations (10^−7^ M – 10^−5^ M), but also at physiological concentrations (10^−10^ M – 10^−8^ M) (Figure 1A). At least 10% of apoptotic 143B cells were observed in the presence of 2-ME ranging from concentrations of 10^−10^ M to 10^−6^ M. While, treatment of 143B OS with 10^−5^ M 2-ME resulted in a dramatic 40% increase in apoptotic cell number in comparison to the control (Figure 1A). Surprisingly, we did not observe any induction of necrosis by physiological relevant concentrations of 2-ME (Figure 1B). Necrosis of 143B OS cells was observed only under pharmacological relevant concentrations (10^−6^ M and 10^−5^ M) of 2-ME (Figure 1B). This is consistent with our previous study [21].

### Effect of physiological and pharmacological relevant concentrations of 2-ME on the inhibition of the 143B OS cell cycle

Previously, our research group demonstrated that 2-ME-induced cell cycle arrest in 143B cells was concentration-dependent [43]. Since physiological and pharmacological relevant concentrations of 2-ME exerted anticancer effects in previous experiments, current studies were performed with the selected, representative concentrations: physiological (10^−8^ M) and pharmacological (10^−7^ M, 10^−6^ M) (Figure 1C). Cell cycle analysis was conducted by imaging cytometry and cells were stained with Hoechst 33342 and Cell Trace™ Calcein Red-Orange. The number of 143B OS cells within the G2 and M phases of the cell cycle increased with a 24 h treatment of every 2-ME concentration (Figure 1C). After 24 h incubation with 10^−8^ M, 10^−7^ M, and 10^−6^ M of 2-ME, 22.16%, 30.15% and 31.53% of the OS cells were in the G2 and M phases, respectively as compared with the control (16.38%). Taken together, 2-ME may also be considered as a physiological oncostatic agent regulating cell proliferation and death.

### Potential mechanisms of 143B OS cell death induced by 2-ME

**Figure 2:**
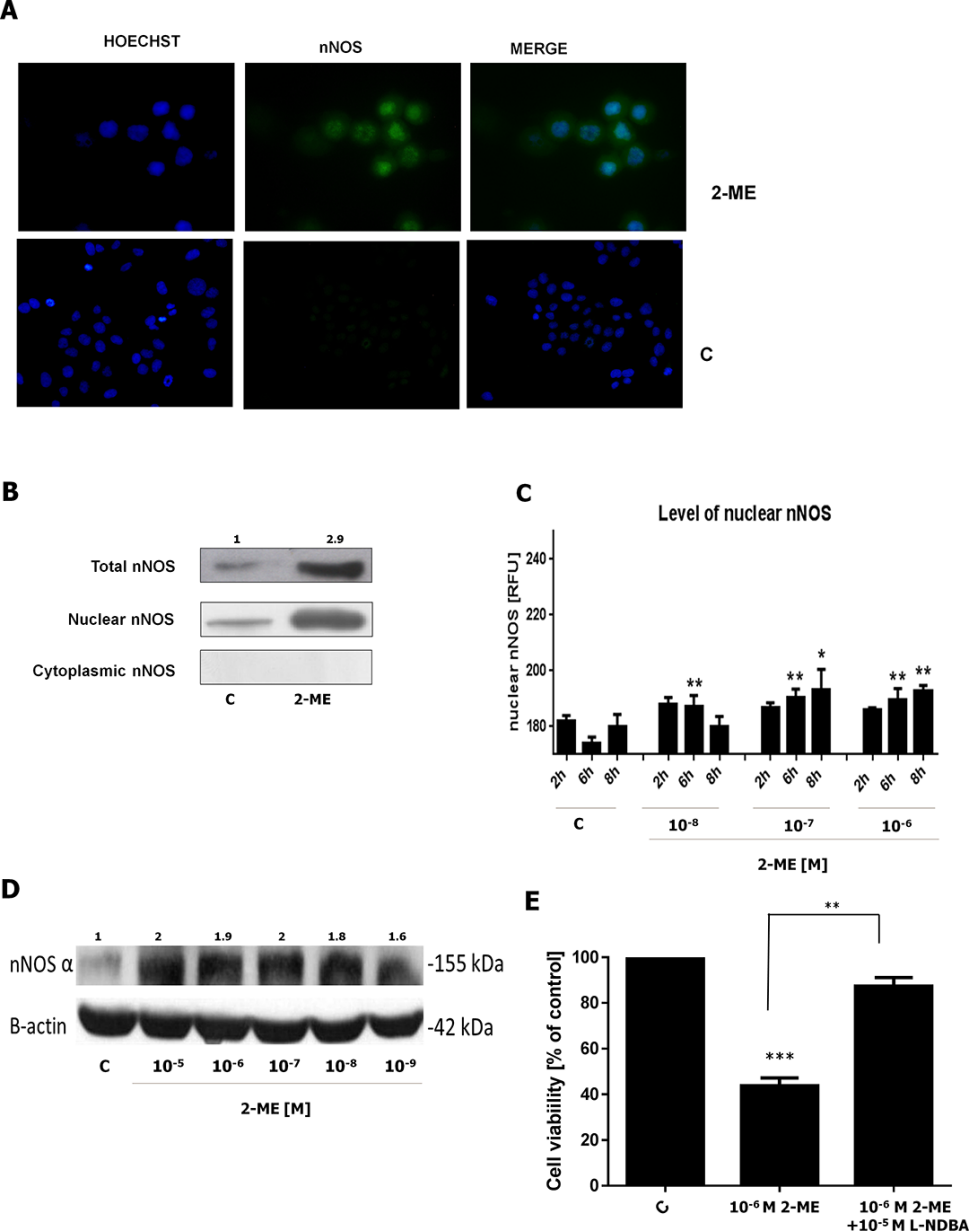
2-ME results in nuclear localization of nNOS in OS 143B cells **A.** 143B OS cells were treated with 10^−6^ M of 2-ME for 8 h. nNOS levels were evaluated by immunofluorescence microscopy. The cells were incubated with a mouse monoclonal antibody against nNOS and a FITC-conjugated, secondary polyclonal rat antibody against mouse IgG (green). Nuclei were visualized with Hoechst 33342 (blue). The representative images are shown. **B.** 143B OS cells were treated with 10^−6^ M of 2-ME for 8 h. Nuclear and cytosolic fractions of nNOS increased by 2-ME were determined by Western blotting. The representative images are shown. **C.** The quantification analysis of nuclear nNOS levels by imaging cytometry. The fluorescence density is presented in RFUs. For nNOS immunostaining, interphase nuclei were used. After 2-, 6-, 8-h treatments with 2-ME (10^−8^ M – 10^−6^ M), 143B OS cells were fixed, then incubated with antibodies (A) The fluorescent signals were captured with an In Cell Analyzer 2000 (GE Healthcare, UK) equipped with a high performance CCD camera. Values are the mean ± SE of three independent experiments (*N* = 6 replicate cultures). The absence of an error bar denotes a line thickness greater than the error. *P < 0.01, **P < 0.001, ***P < 0.0001, ****P < 0.00001 versus control cells (C). **D.** 1 h treatment of 143B OS cells with 2-ME (10^−9^ M −10^−5^ M) resulted in an increase of the total level of nNOSα. The result was obtained by Western blotting using nNOS specific antibodies. The band corresponding to nNOS alpha was analyzed. The representative data are shown. **E.** 10^−5^ M L-NDBA reversed 2-ME-induced inhibition of 143B OS cell growth. Values are the mean ± SE of three independent experiments (*N* = 6 replicate cultures). The absence of an error bar denotes a line thickness greater than the error. *P < 0.01, **P < 0.001, ***P < 0.0001, ****P < 0.00001 versus control cells (C) Each experiment was performed at least three times.

In our previous study, we demonstrated that 2-ME under high pharmacological relevant concentrations selectively increased nNOS level within 143B OS and HT22 hippocampal cells, resulting in cell death [21]. The maximal nNOS expression peaked between 6 h and 8 h of incubation. Moreover, specific inhibitor of nNOS reversed cell death. E2 (10^−6^ M, 10^−5^ M) did not significantly affect nNOS protein levels [21]. Thus, this mode of action seems to be unique for 2-ME, since E2 did not elicit any effects on nNOS concentrations [21]. Similar effects were found at physiological concentrations of 2-ME (Figure 2).

In the current study, nNOSα expression was analyzed by Western blotting using specific anti-nNOS antibodies (Figure 2D). nNOSα was reported as an isoform that may be recruited to the nucleus [26, 27, 28]. As presented, total level of nNOSα increased after 1 h of incubation with 10^−9^ M to 10^−5^ M of 2-ME (Figure 2D). Precisely, nNOSα increased 2-fold after incubation with 10^−7^ M to 10^−5^ M of 2-ME. Incubation with 10^−8^ M and 10^−9^ M of 2-ME altered nNOSα expression 1.84 and 1.64 times, respectively. Immunofluorescence microscopy and imaging cytometry was used to analyze the intracellular localization of nNOS in 143B OS cells. Physiological (10^−8^ M) and pharmacological (10^−7^ M, and 10^−6^ M) concentrations of 2-ME were used. We observed that nuclear fraction of nNOS was elevated after stimulation with all used concentrations of 2-ME (Figure 2A–2C). Significant increases were observed in the level of nNOS in nuclei of 143B cells after 2 h and 6 h of incubation with 10^−8^ M 2-ME. Treatment with pharmacological relevant concentrations (10^−7^ M, and 10^−6^ M) resulted in nuclear nNOS localization after 6 h and 8 h of incubation (Figure 2A). These data are in agreement with the observed increase in the total level of nNOSα (Figure 2C). Inhibition of 143B OS cell growth by 10^−6^ M of 2-ME was significantly reversed after adding L-NDBA, an nNOS inhibitor (*N*ω-Nitroarginine-2,4-L-diaminobutyric amide di(trifluoroacetate) salt 10^−5^ M) (Figure 2E).

### Nitro-oxidative stress level after stimulation with 2-ME

**Figure 3:**
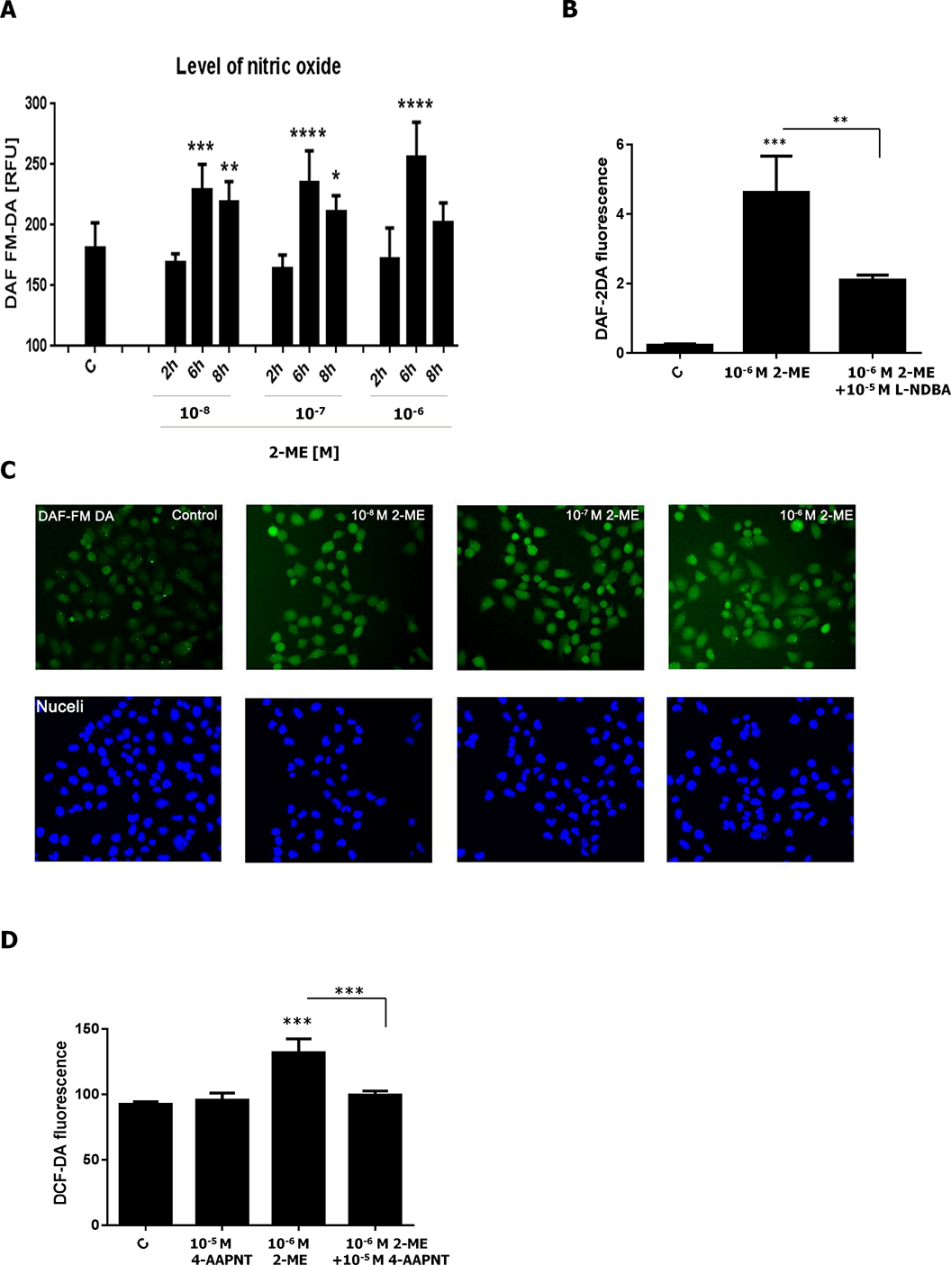
Induction of nitro-oxidative stress by 2-ME **A.** NO production increased by 2-ME after 2-, 6-, 8-h treatments with 2-ME (10^−8^ M – 10^−6^ M). NO levels were evaluated by imaging cytometry using fluorogenic probes, DAF-FM DA (5 × 10^−6^ M), and a 10-min incubation time in PBS buffer. The fluorescent signals were captured with an In Cell Analyzer 2000 (GE Healthcare, UK) equipped with a high performance CCD camera. The fluorescence density is presented in RFUs. Values are the mean ± SE of three independent experiments (*N* = 6 replicate cultures). The absence of an error bar denotes a line thickness greater than the error. *P < 0.01, **P < 0.001, ***P < 0.0001, ****P < 0.00001 versus control cells (C). **B.** NO production induced by 2 h incubation with 10^−6^ M of 2-ME was reversed by specific nNOS inhibitor, L-NDBA (10^−5^ M). **C.** Representative images of induction of NO by 2-ME determined by imaging cytometry (A) Cell nuclei are shown in blue and the nitric oxide levels in green. The fluorescent signals were captured with an In Cell Analyzer 2000 (GE Healthcare, UK) equipped with a high performance CCD camera. **D.** Nitro-oxidative stress induced by an 8 h incubation with 10^−6^ M of 2-ME was reversed by specific nNOS inhibitor, 4-AAPNT (10^−5^ M). Values are the mean ± SE of three independent experiments (*N* = 6 replicate cultures). The absence of an error bar denotes a line thickness greater than the error. *P < 0.01, **P < 0.001, ***P < 0.0001, ****P < 0.00001 versus control cells (C) Each experiment was performed at least three times.

Previously, we demonstrated using flow cytometry and 4,5-diaminofluorescein diacetate (DAF-2DA) indicator that incubation of HT22 hippocampal and 143B OS cells with 10^−6^ M 2-ME resulted in an increase in NO levels due to the upregulation of nNOS [21]. Herein, NO production was measured after treatment of 143B cells with physiological and pharmacological relevant concentrations of 2-ME. Imaging cytometry and specific fluorescent indicators were used to determine the NO level in live cells. 4-amino-5-methylamino-2′, 7′-difluorofluorescein diacetate (DAF-FM DA) was chosen as the indicator because it is more sensitive to NO, more photo-stable and less pH sensitive than DAF-2DA [63, 64]. A significant increase in the level of NO was observed in a time-dependent manner (Figure 3A). After 2 h of incubation with 10^−8^ M, 10^−7^ M, and 10^−6^ M of 2-ME, the level of NO was only slightly augmented by 3.4%, 1%, 5.5% in comparison to the control, respectively (Figure 3A). NO level significantly increased by 24%, 27%, 39% after 6 h of treatment with 10^−8^ M, 10^−7^ M, 10^−6^ of 2-ME, respectively (Figure 3A, 3C). Interestingly, after 8 h of incubation, an increase in NO levels was detected only after treatment with 10^−8^ M and 10^−7^ M of 2-ME (Figure 3A, 3C). A 2-ME-mediated increase in the level of NO was observed without the presence of the nNOS inhibitor, L-NDBA. As demonstrated in Figure 3B, level of NO was increased by 10^−6^ M 2-ME and then significantly decreased after preincubation with 10^−5^ M L-NDBA. Induction of nitro-oxidative stress determined by 2′,7′-dichloro-fluorescein diacetate (DCF-DA) fluorescence by 10^−6^ M 2-ME was also reversed by using another specific nNOS inhibitor, 4-AAPNT (*N*-[(4*S*)-4-amino-5-[(2-aminoethyl)amino]pentyl]-*N*′-nitroguanidine tris(trifluoroacetate), 10^−5^ M) (Figure 3D). These results confirmed the involvement of nNOS in the anticancer mechanisms of action of 2-ME. Increased expression of nNOS was correlated with enhanced production of NO (Figure 2A, 2C).

### 2-ME-induced DNA damage

**Figure 4:**
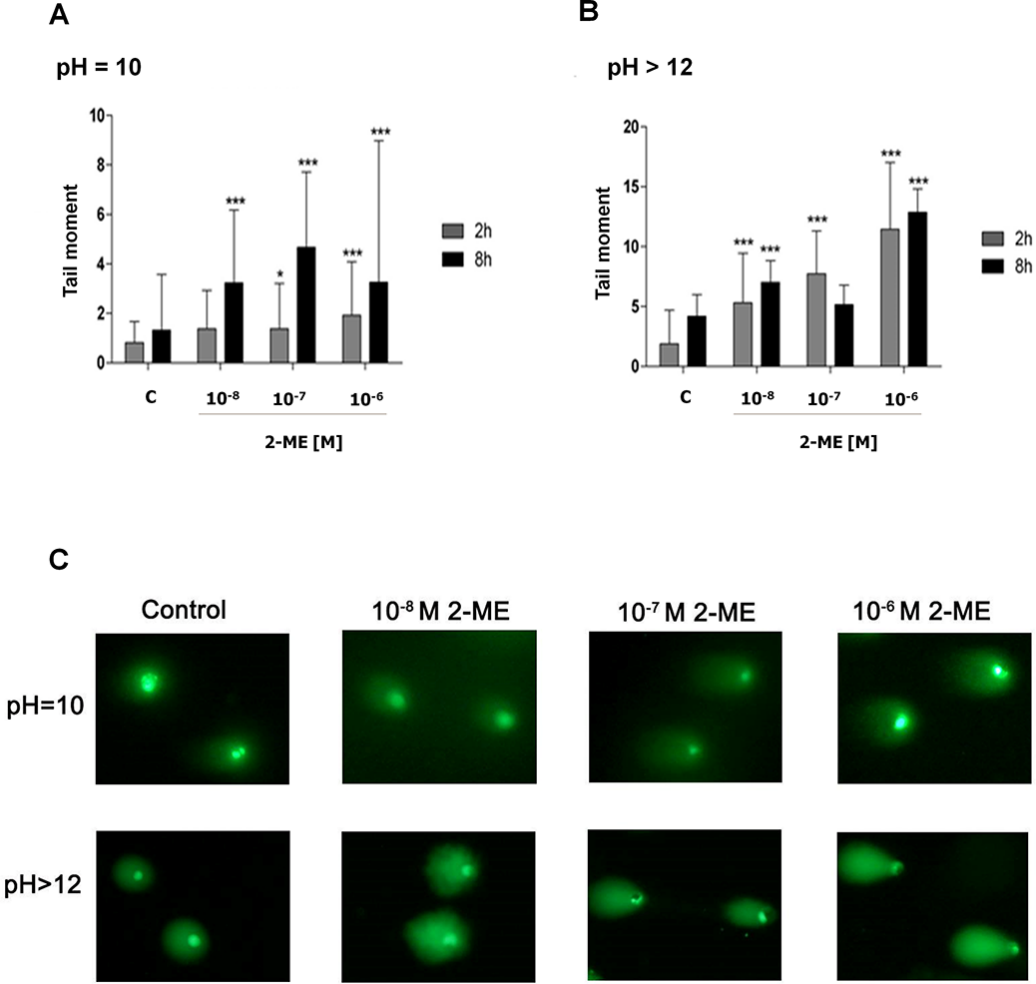
2-ME induced DNA damage at physiological and pharmacological relevant concentrations **A, B.** DSBs and SSBs after 2- and 8-hour treatments with 2-ME (10^−8^ M – 10^−6^ M) determined by (pH = 10) and alkaline (pH > 12) comet assay, respectively. The tail moment was considered a general parameter to the DNA integrity assessment. Values are the mean ± SE of three independent experiments. 100 single cells were taken into analysis of DNA damage. The absence of an error bar denotes a line thickness greater than the error. *P < 0.01, **P < 0.001, ***P < 0.0001, ****P < 0.00001 versus control (C). **C.** DNA damage measurements at neutral and alkaline pH after a 2 h treatment with 2-ME (10^−8^ M – 10^−6^ M). The representative images are shown. Each experiment was performed at least three times.

**Figure 5:**
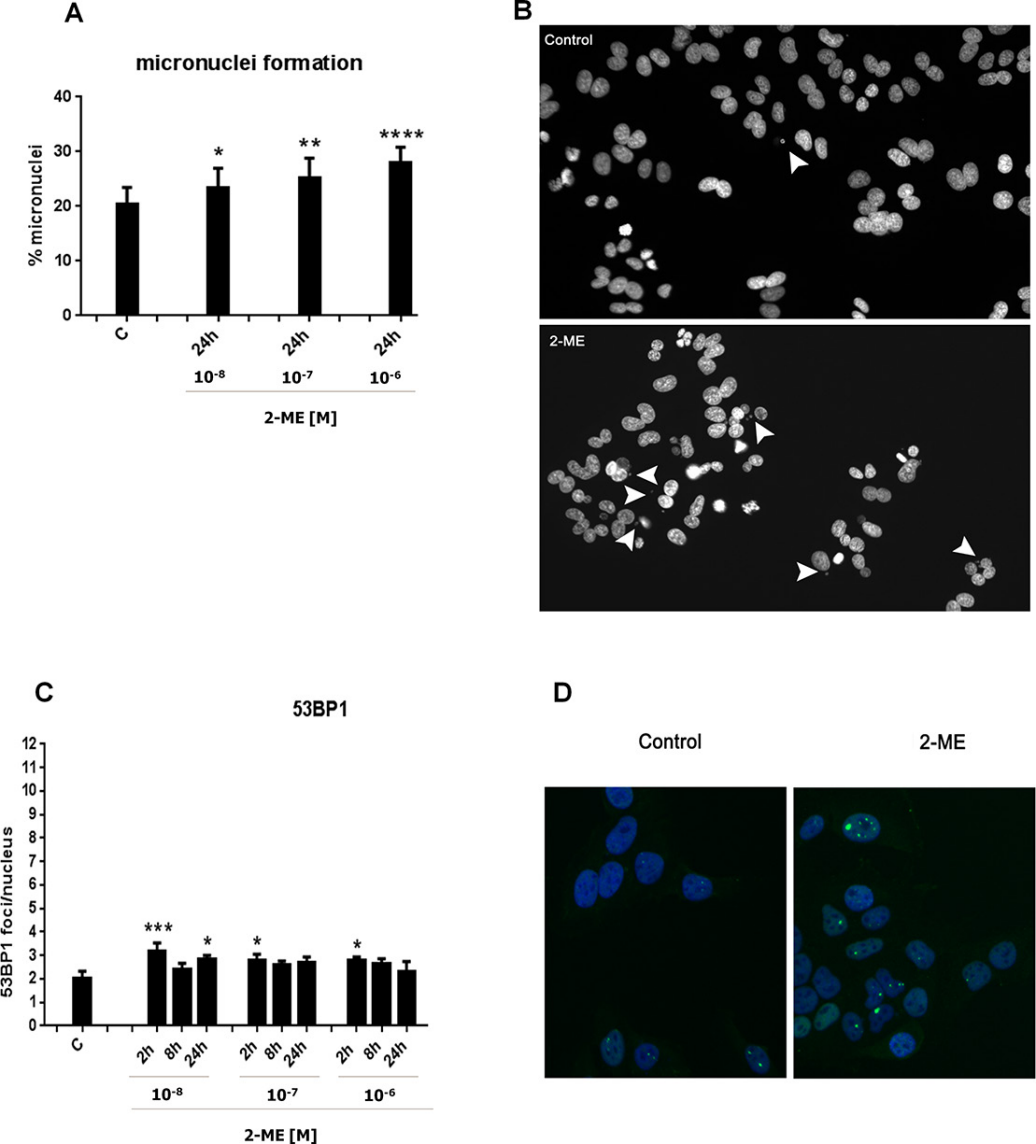
2-ME induced genomic instability and DNA repair at physiological and pharmacological relevant concentrations **A, B.** Micronuclei formation after the 24 h treatment with 2-ME (10^−8^ M – 10^−6^ M) determined by CBMN assay using imaging cytometry. Arrowheads indicate micronuclei. **C, D.** 143B OS cells were treated with 2-ME (10^−8^ M – 10^−6^ M) for 2, 8, 24 h and 2-ME stimulated DNA repair by increasing 53BP1 foci/nucleus (green) was investigated. Nuclei were visualized using Hoechst 33342 staining (blue). Values are the mean ± SE of three independent experiments (*N* = 6 replicate cultures). 500 single cells were taken into analysis of DNA damage and DDR. The representative images are shown (B, D). The absence of an error bar denotes a line thickness greater than the error. *P < 0.01, **P < 0.001, ***P < 0.0001, ****P < 0.00001 versus control (C). Each experiment was performed at least three times.

We were wondering whether 2-ME increased nNOS levels and nitro-oxidative stress generation within the nuclei of 143B OS cells may exert any effects on DNA damage and genomic instability In a previous study, the nNOS inhibitor, L-NDBA, significantly decreased the DNA fragmentation rate after 16 h of incubation with 10^−6^ M of 2-ME [21 and Figure 5B]. Due to the fact that we observed nNOS induction after 1 h maintaining till 8 h of incubation with both physiological and pharmacological concentrations of 2-ME (Figure 2C), we assessed DNA strand breaks after 2 h and 8 h of incubation with 2-ME. Two versions of the comet assay were used to evaluate DNA damage. The alkaline assay detected single strand breaks (SSBs) and the neutral assay detected double strand breaks (DSBs) [39]. Time- and 2-ME-concentration-dependent increases in both SSBs and DSBs were observed (Figure 4). We present that 2-ME increased DNA damage as soon as after 2 h of incubation with the stimuli what is in the correlation with increased total nNOS level (Figure 2). DSBs were 1.37, 1.37, and 1.92 times greater in number after 2 h of incubation with 10^−8^ M, 10^−7^ M, and 10^−6^ M of 2-ME in comparison to the control (0.82), respectively (Figure 4A). Incubation with 10^−8^ M, 10^−7^ M, and 10^−6^ M of 2-ME for 2 h resulted in 5.30-, 7.72-, and 11.45- fold increases in the SSBs in comparison to the control (1.86), respectively (Figure 4B, 4C). The number of DSBs and SSBs subsequently increased after 8 h of incubation with 2-ME. In comparison to the control (1.31), 3.23-, 4.66-, and 3.25-fold increases were observed in DSBs after 143B OS cells were incubated with 10^−8^ M, 10^−7^ M, and 10^−6^ M of 2-ME for 8 h, respectively (Figure 4A, 4C). SSBs increased by a factor of 7.02, 5.14, and 12.85 after 8 h of incubation with 10^−8^ M, 10^−7^ M, and 10^−6^ M of 2-ME in comparison to the control (4.18), respectively (Figure 4B, 4C). Appearance of SSBs and DSBs after 2 h of incubation with 2-ME seemed to be one of the first signals of DNA damage. The cytokinesis-block micronucleus (CBMN) assay confirmed that 2-ME stimulated genotoxicity in 143B OS cells. Micronuclei formation is correlated with altered genomic stability. Genomic instability is often associated with cancer and may be indicative of a poor prognosis for some types of cancer [74, 75, 76, 77]. By imaging cytometry the influence of 2-ME on micronucleus formation was examined. Our experimental data suggested that a time period of 24 h was sufficient for the effective cell division and micronucleus formation of 143B OS cells. 24 h treatment with physiological and pharmacological relevant concentrations of 2-ME lead to increased genomic instability within the 143B cells. This confirmed the genotoxic effects of 2-ME within the experimental model. Incubation with 2-ME resulted in an increased amount of micronuclei that was concentration-dependent. After 24 h of incubation with 10^−8^ M, 10^−7^ M, and 10^−6^ M of 2-ME, micronucleus formation increased by 23.4% ± 1.5, 25.3% ± 1.4, and 28% ± 1.1 in comparison to the control (20.35% ± 1), respectively (Figure 5A, 5B). Activation of the DNA damage response (DDR) is an important determinant of cell sensitivity to chemotherapeutic drugs eliminating tumor cells. 53BP1 is a protein recognizing the central DNA-binding domain of p53. It relocates to the sites of DNA strand breaks in response to DNA damage [39]. We investigated whether the DNA damage response was activated after the treatment of OS cells with 2-ME at physiological and pharmacological concentrations. DNA damage detected as 53BP1 foci/nucleus was evaluated by means of imaging cytometry. As demonstrated in Figure 5, recruitment of 53BP1 was significantly increased after 2 h of treatment at all concentrations of 2-ME (Figure 5C, 5D). The recorded data fully supported the observed SSBs and DSBs in 143B OS cells after treatment with 2-ME (Figure 4). The cells in which DDR was activated may be those that did not undergo cell death, another will be transferred to the way of cell death [78].

## DISCUSSION

Herein, we demonstrated a new mode of anticancer action of 2-ME at physiological and pharmacologically relevant concentrations. Increased levels of nNOS within the nuclei of 143B OS cells and the subsequent induction of nitro-oxidative stress caused cell cycle arrest and cancer cell death. We used the OS experimental model due to its high malignancy, chemoresistance and genomic instability [101].

Various preclinical and clinical cancer models proved the anticancer properties of 2-ME [13, 14, 15, 48, 49, 50, 51, 52], though there are only few reports concerning the physiological activity of 2-ME [5, 6, 53]. Similarly to our current results, Vijayanathan and co-workers demonstrated the inhibitory effects of 10^−8^ M of 2-ME towards MCF-7 cell growth [5]. Decreased concentrations of 2-ME have been implicated as one of the reasons for pre-eclampsia, suggesting that 2-ME plays an important physiological role [6]. For the first time, we managed to prove that at physiological 2-ME concentrations (10^−10^ M – 10^−8^ M) induced apoptosis in highly metastatic 143B OS cells. While high pharmacological relevant 2-ME concentrations (10^−6^ M – 10^−5^ M) induced necrosis. Little information is available concerning the induction of necrosis within cancer cells by 2-ME. High pharmacological concentrations of 2-ME induced necrosis within human endometrial carcinoma cells (RL-95–2) [44]. Up to date, 2-ME was shown to inhibit cell growth and induce apoptosis or cell death in cancer cells [19, 21, 33, 35, 45, 54–56].

Previously, we demonstrated that high pharmacological concentrations of 2-ME lead to OS and hippocampal cell death through the induction of nNOS [21]. In the current study, we hypothesize that, under physiological and therapeutical conditions, 2-ME stimulates the nuclear hijacking of nNOS and directly induced nitro-oxidative stress within the nucleus. 2-ME seems to be strictly associated with pro-apoptotic and oncostatic mechanisms of action. nNOS has been believed to be a cytosolic isoform [57, 58]. However, different splicing variants and subcellular localizations of the enzyme were recently reported [26]. Nonetheless, the exact role of nNOS within the nucleus has continued to remain undefined [26, 27, 59]. The effect of 2-ME on proteins regulating nNOS translocation from the cytosol to the nucleus is currently under our investigation. In contrast to eNOS or iNOS, nNOS is a larger protein containing a PDZ domain at its N-terminal, a consensus sequence of approximately 90 amino acids [60–62]. The PDZ domain seems to be an important factor for the facilitation of nNOS to distinct intercellular compartments [27, 59]. Aquilano and colleagues demonstrated that PDZ is essential for the nuclear recruitment of nNOS, thus favoring NO production [26]. 2-ME-increased nitro-oxidative stress associated with the generation of NO and/or its derivatives within the nuclei of OS cells may have directly resulted in SSBs and DSBs, leading to 143B OS cell death. The link between nuclear proteotoxic stress and cancer cell death has been recently reported [79, 80, 81]. Local generation of NO and/or their reactive derivatives (nitrogen dioxide, peroxynitrite) are likely contributed to DNA damage [82, 83, 84]. *In vitr*o studies with cellular models indicated that NO and its derivatives were able to induce direct- and mediated-genotoxic effects [85, 86]. Bossy-Wetzel and Lipton reported that excessive amounts of NO may have induced S-nitrosylation and/or triggered DNA damage, resulting in cell death [87]. ROS/RNS action on DNA may have caused several modifications of nucleotides and generated SSBs and DSBs [82, 84, 88]. Oxidative stress preferentially induced micronucleus formation and mediated the genomic instability caused by p53 dysfunction [89]. Diverse chemotherapeutics like 2-ME may be selectively toxic to tumor cells by increasing oxidative/nitro-oxidative stress and pushing the already-stressed cancer cells beyond their limit [90, 91]. DNA damage caused by increased nNOS levels within OS cell nuclei led to cancer cell death at physiological and pharmacological concentrations of 2-ME. Consistent with our study, 2-ME was reported to enhance the sensitivity of glioma cell lines to radiotherapy by arresting the cell cycle at the G2/M phase and increased DNA damage [92]. Khoei and colleagues demonstrated that combined treatment with 25 × 10^−5^ M of 2-ME and ^60^Co significantly increased iododeoxyuridine DNA damage. Only slight DNA damage was observed when 2-ME was administered separately [93]. This result may be attributable to the U87MG glioblastoma cancer cell line spheroid model. Higher resistance has been observed within spheroid cultures in comparison to monolayer cultures [93, 94]. Moreover, cancer cell chemosensitization by NO during anticancer therapy has been reported [95]. Treatments of human cancer cells with NO and NO mimetics also have been shown to restore chemoresistance both *in vivo* and *in vitro* [96, 97, 98, 99]. On the other hand, malfunction of DNA damage repair system may result in chemoresistance of cancer cells and further cancer progression [75, 78, 100]. It may be considered as one of the limitations of long-term 2-ME use.

**Figure 6:**
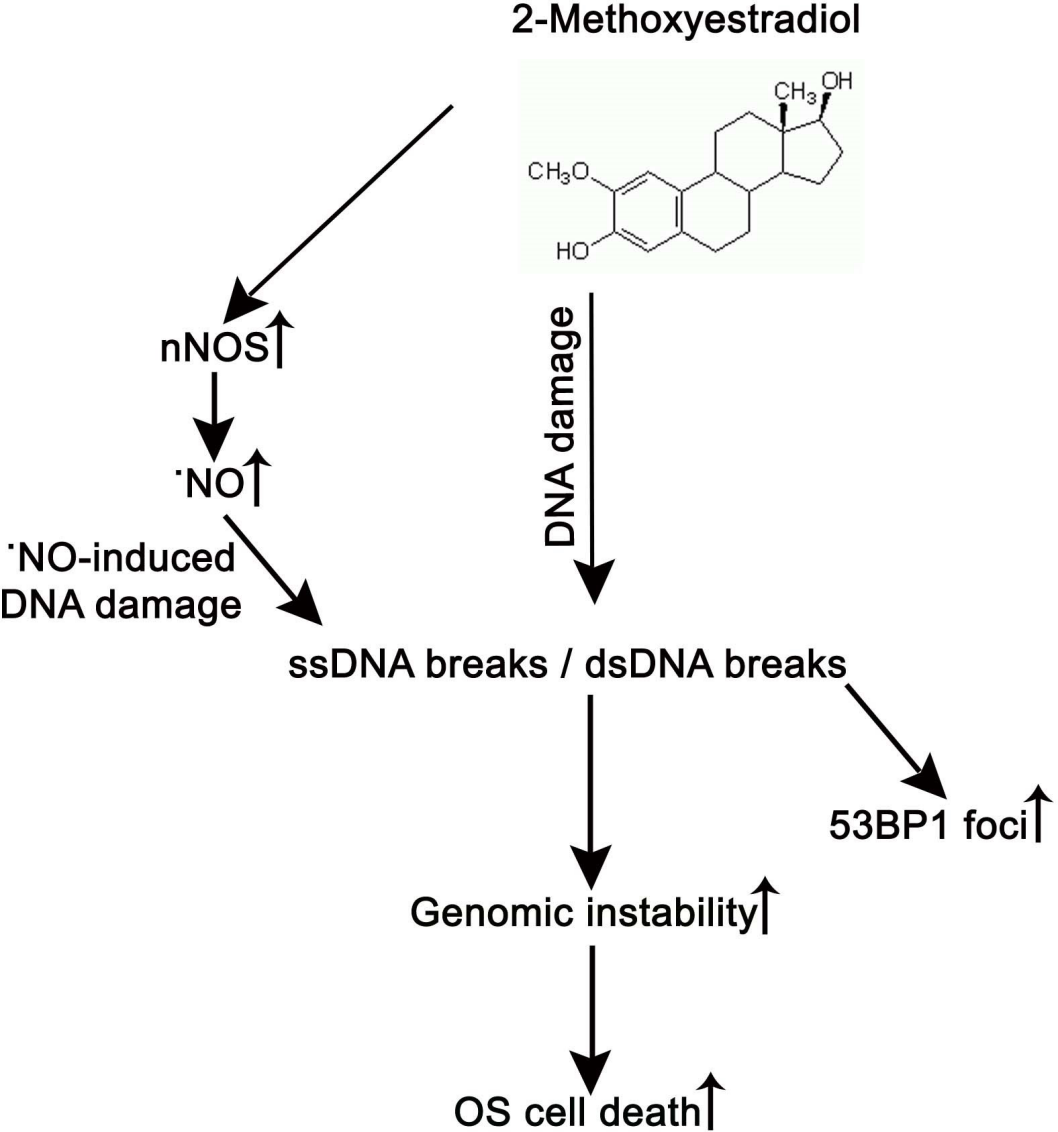
2-ME induces OS cell death via increasing nuclear nNOS resulting in DNA damage and genomic instability

In conclusion, we demonstrated that SSBs and DSBs is involved in the 2-ME oncostatic mechanism (Figure 6). Anticancer effects were observed at physiological and pharmacologically relevant concentrations. Mechanisms of nuclear transport of nNOS induced by 2-ME need to be further investigated.

## MATERIALS AND METHODS

### Cell line and culture conditions

143B OS cell line was obtained from the American Tissue Type Collection (ATTC-8303). Cells were cultured at 37°C in a humidified atmosphere and saturated with 5% CO_2_. Dulbecco's Modified Eagle's Medium was supplemented with 10% heat-inactivated fetal bovine serum and a penicillin (100 μg/mL)/streptomycin (100 μg/mL) cocktail (Sigma-Aldrich, Poland).

### Cell treatment

The 143B OS cells were treated with various concentrations of 2-ME and time, depending on the design of experiments. 10^−5^ M of nNOS inhibitor (4-AAPNT, L-NDBA) was added to the cells after 2 h of pre-treatment. The medium was removed and the cells were washed with a phosphate-buffered saline (PBS, Sigma Aldrich). A new medium containing 2-ME replaced the previous one. We used charcoal-stripped FBS (Sigma Aldrich, Poland) in the medium for cell treatment. Charcoal-stripped FBS is used to elucidate the effects of hormones in a variety of *in vitro* systems.

### Reagents

Tissue culture media, antibiotic cocktail, fetal bovine sera, 2-ME, the nNOS inhibitors L-NDBA, 4-AAPNT were purchased form Sigma Aldrich Poland. Anti-rabbit IgG were purchased from Abcam (Cambridge, UK). Mouse antibodies against nNOS, secondary polyclonal rat antibody against mouse IgG were obtained from BD Biosciences (Germany).

### Assessment of apoptosis by flow cytometry with Annexin V-propidium iodide (PI) staining

The analysis was performed as previously described [21, 36]. 143B OS cells were seeded onto six-well plates at a density of 3 × 10^5^ cells/well. After 24 h of culturing in the standard medium, the cells were treated with 2-ME for 24 h. The cells were then pelleted and incubated with Annexin V and PI according to manufacturer's protocol (BD Pharmingen, Poland). Afterwards, the cells (3 × 10^4^/sample) were analyzed and the fluorescent signals of Annexin V conjugate and PI were detected at the fluorescence intensity channels FL1 and FL3 (BD FACScan). The results were then analyzed by Cyflogic software, version 1.2.1. Each experiment was performed at least three times.

### Cell cycle analysis

After treatment with 2-ME, 143B OS cells were stained with a mixture of Hoechst 33342 (2.5 μg/ml) and Cell Trace™ Calcein Red-Orange AM (2.5 × 10^−6^ M) (Life Technologies, Poland) in a serum-free DMEM medium at 37°C for 30 min. The OS cells were rinsed with PBS and subjected to cell cycle analysis using an In Cell Analyzer 2000 (GE Healthcare, UK) equipped with a high performance CCD camera.

### Assessment of nitro-oxidative stress by flow cytometry

ROS and RNS production were determined using flow cytometry with DCF-DA staining. The cells were seeded onto six-well plates at a density of 3 × 10^5^ cells/well. After 24 h of culturing in the normal growth medium, cells were exposed to treatment with 2-ME for 24 hours. Subsequently, 30 min before the end of the incubation time, a solution of DCF-DA was added to each well of treated cells in order to obtain the final concentration of 10^−5^ M for 2-ME. Next, both floating and trypsinized cells were collected by centrifugation at 1200 rpm for 7 min. The pellet was next washed twice with PBS, followed by resuspension in PBS. The whole procedure was performed on ice. Afterward, 3 × 10^4^ cells were analyzed by flow cytometry (BD FACScan) and the results were analyzed by Cyflogic software, version 1.2.1. Each experiment was performed at least three times.

### NO level by imaging cytometry

Imaging of RNS production in living cells based on fluorescent indicators has been reported as a fast, sensitive and selective method [40, 41]. After 2-, 6-, 8-h treatments with 2-ME, NO levels were evaluated using the fluorogenic probe DAF-FM DA (5 × 10^−6^ M) and a 10-min incubation time in a PBS buffer. NO-specific fluorescent signals were captured with an In Cell Analyzer 2000 (GE Healthcare, UK) equipped with a high performance CCD camera. For NO positive control, a NO donor – MAHMA NONOate (10^−3^ M) was used.

### Immunofluorescence microscopy

The immunofluorescence was performed as previously described [50, 21]. The 143B OS cells were treated with 2-ME for 8 h. Anti-nNOS (1:50 in 0.3% GSA, 2 h incubation, BD Biosciences) and goat anti-mouse secondary-conjugated with CY3 (1:100, GAM Cy3, 1 h incubation, Jackson Immunoresearch, Suffolk, UK) antibodies were used. The images were analysed and merged employing the ImageJ software 1.44p. Each experiment was performed at least three times.

### nNOS immunostaining by imaging cytometry

For nNOS immunostaining, interphase nuclei were used. After 2-, 6-, and 8-h treatments with 2-ME in the 96-well plate, 143B OS cells were fixed with 3.7% formaldehyde containing 0.1% Triton X-100 in PBS for 20 min. Subsequently, the cells were incubated with 1% bovine serum albumin (BSA) in PBST (phosphate buffered saline containing 0.25% Triton X-100) at room temperature for 30 min. After washing with PBST, the cells were incubated with mouse monoclonal antibodies against nNOS (BD Biosciences, Germany) (diluted 1:50 in PBST–BSA [PBST containing 1% BSA]) overnight at 4°C. The next day, FITC-conjugated, secondary polyclonal rat antibodies against mouse IgG (BD Biosciences, Germany) (diluted 1:1000 in PBST–BSA) were added and incubated at room temperature for 1 h. Nuclei were visualized with Hoechst 33342. Digital cell images were captured with an In Cell Analyzer 2000 (GE Healthcare, UK) equipped with a high performance CCD camera. To analyze cellular nNOS content and localization, In Cell Analyzer software (In Cell Analyzer Investigator) was used. The fluorescence density was presented in relative fluorescence units (RFUs). As a positive control, treatment with 0.1 mg/ml nocodazole was used.

### 53BP1 immunostaining

The analysis was performed as previously described [39]. For 53BP1 immunostaining, interphase nuclei were used. After 2-, 8- and 24-h treatments with 2-ME in the 96-well plate, 143B OS cells were fixed with 3.7% formaldehyde containing 0.1% Triton X-100 in PBS for 20 min. Subsequently, the cells were incubated with 1% bovine serum albumin (BSA) in PBST (phosphate buffered saline containing 0.25% Triton X-100) at room temperature for 30 min. After washing with PBST, the cells were incubated with a rabbit polyclonal antibody against 53BP1 (Novus Biologicals, Poland) (diluted 1:200 in PBST–BSA (PBST containing 1% BSA)) overnight at 4°C. The next day, FITC-conjugated secondary polyclonal antibodies against rabbit IgG (BD Biosciences, Germany) (diluted 1:200 in PBST–BSA) were added and incubated at room temperature for 1 h. Nuclei were visualized with Hoechst 33342. Digital cell images were captured with an In Cell Analyzer 2000 (GE Healthcare, UK) equipped with a high performance CCD camera. 53BP1 foci were scored per nucleus.

### Comet assay

The analysis was performed as previously described [39]. DNA double-strand breaks (DSBs) and DNA single-strand breaks (SSBs) were assessed with neutral and alkaline single-cell microgel electrophoresis (comet assay), respectively. After 2- and 8-h treatments with 2-ME, 143B OS cells were suspended in PBS and mixed with low melting (LM) agarose (0.7%). The cells were fixed to agarose (LM) slides and lyzed with proteinase K (0.5 mg/ml) and reduced glutathione (2 mg/ml) in a lysis solution (1.25 M NaCl, 50 mM EDTA, 100 mM Tris–HCl, 0.01% N-lauroylsarcosine sodium salt, pH 10) at 37°C for 2 h. Electrophoresis (neutral comet assay buffer: 100 mM Tris–HCl, 0.5 M NaCl, 1 mM EDTA, 0.2% DMSO, pH 10 and alkaline comet assay buffer: 1 mM EDTA, 0.2% DMSO, 300 mM NaOH, pH > 12) was performed on the treated cells. The slides were then stained with 2.5 × 10^7^ M YOYO-1 (Invitrogen Corporation, Grand Island, NY, USA) in a 2.5% DMSO and 0.5% sucrose solution. The cells were mounted with a coverslip and digital comet images were immediately captured with an Olympus BX61 fluorescence microscope equipped with a DP72 CCD camera and Olympus CellF software. The CCD capture conditions were: exposure time 81 ms, magnification 400x. Images were saved as TIFF files. At least 100 comets were measured per each sample triplicate using AutoComet Software http://autocomet.com/index.php (TriTek Corp). The Tail Moment (Tail moment = tail length × fraction of total DNA in the tail) was scored as general parameter to DNA integrity assessment.

### Cytokinesis-block micronucleus (CBMN) assay

143B OS cells were treated with 2-ME for 24 h. Micronucleus generation was measured with a CBMN assay using the BD™ Gentest Micronucleus Assay Kit and following the standard protocol outlined in the manufacturer's instructions. A total of 500 binucleated cells per well [37, 38] were scored using an In Cell Analyzer 2000 (GE Healthcare, UK) equipped with a high performance CCD camera. For a positive control, 24 h treatment with 100 ng/ml mitomycin C was used.

### Western blotting

The cytoplasmic and nuclear fractions were separated using the Nuclear Extract Kit (Active Motif, France) according to manufacturer's protocol. Equal amounts of total cell lysates were resolved by 7% SDS-PAGE. The membranes were then incubated with primary antibodies anti-nNOS (BD Biosciences) (1:1000) overnight at 4°C and an analysis was performed as previously described [21, 36]. The chemiluminescence was detected using ImageQuant LAS 500 (GE Healthcare). The protein level was quantified by densitometry technique using the Quantity one 4.5.2 software. The protein levels, as determined by chemiluminescent signal quantification, were normalized relative to beta-actin levels found in the samples. Each experiment was performed at least three times.

### Statistical analysis

The results represent the mean ± SD from at least three independent experiments. All microscopic evaluations were done on randomized and coded slides. Differences between control samples versus 2ME-treated samples were assessed with one-way analysis of variance (ANOVA) with *post hoc* testing using a Dunnett's multiple comparison test. A *p*-value of less than 0.01 was considered to correspond with statistical significance. Data were analyzed using GraphPad Prism (GraphPad Software, Inc., version 6, USA).

## Abbreviations

4-AAPNT: *N*-[(4*S*)-4-amino-5-[(2-aminoethyl)amino]pentyl]-*N*′-nitroguanidine tris(trifluoroacetate)
C: control
DAF-2DA: 4,5-diaminofluorescein diacetate
DAF-FM DA: 4-Amino-5-methylamino-2′,7′-difluorofluorescein diacetate
DCF-DA: 2′,7′-dichloro-fluorescein diacetate
DDR: DNA damage response
DHE: dihydroethidium
DSBs: DNA double-strand breaks
E2: 17β-estradiol
2-ME: 2-methoxyestradiol
L-NDBA: *N*ω-Nitroarginine-2,4-L-diaminobutyric amide di(trifluoroacetate) salt
OS: osteosarcoma
RFUs: relative fluorescence units
ROS: reactive oxygen species
RNS: reactive nitrogen species
SOD: superoxide dismutase
SSBs: DNA single-strand breaks
